# Synthesis of Mesoporous TiO_2_/Boron-Doped Diamond Photocatalyst and Its Photocatalytic Activity under Deep UV Light (λ = 222 nm) Irradiation

**DOI:** 10.3390/molecules23123095

**Published:** 2018-11-27

**Authors:** Norihiro Suzuki, Akihiro Okazaki, Haruo Kuriyama, Izumi Serizawa, Aiga Hara, Yuiri Hirano, Yukihiro Nakabayashi, Nitish Roy, Chiaki Terashima, Kazuya Nakata, Ken-ichi Katsumata, Takeshi Kondo, Makoto Yuasa, Akira Fujishima

**Affiliations:** 1 Photocatalysis International Research Center, Research Institute for Science and Technology, Tokyo University of Science, 2641 Yamazaki, Noda, Chiba 278-8510, Japan; asdfghjk@tmu.ac.jp (Y.N.); nitu.iitg@gmail.com (N.R.); terashima@rs.tus.ac.jp (C.T.); nakata@rs.tus.ac.jp (K.N.); k.katsumata@rs.tus.ac.jp (K.-i.K.); fujishima_akira@rs.tus.ac.jp (A.F.); 2 ORC Manufacturing Co., Ltd., 4896 Tamagawa, Chino, Nagano 391-0011, Japan; a-okazaki@orc.co.jp (A.O.); h-kuriyama@orc.co.jp (H.K.); i-serizawa@orc.co.jp (I.S.); 3 Faculty of Science and Technology, Tokyo University of Science, 2641 Yamazaki, Noda, Chiba 278-8510, Japan; 7216658@alumni.tus.ac.jp (A.H.); 7216661@alumni.tus.ac.jp (Y.H.); t-kondo@rs.noda.tus.ac.jp (T.K.); yuasa@rs.noda.tus.ac.jp (M.Y.)

**Keywords:** surfactant-assisted sol-gel method, mesoporous metal oxide, thin film, photocatalyst, p-n heterojunction, water purification, deep UV light

## Abstract

There is a need for highly efficient photocatalysts, particularly for water purification. In this study, we fabricated a mesoporous TiO_2_ thin film on a boron-doped diamond (BDD) layer by a surfactant-assisted sol-gel method, in which self-assembled amphiphilic surfactant micelles were used as an organic template. Scanning electron microscopy revealed uniform mesopores, approximately 20 nm in diameter, that were hexagonally packed in the TiO_2_ thin film. Wide-angle X-ray diffraction and Raman spectroscopy clarified that the framework crystallized in the anatase phase. Current–voltage (I–V) measurements showed rectification features at the TiO_2_/BDD heterojunction, confirming that a p–n hetero-interface formed. The as-synthesized mesoporous TiO_2_/BDD worked well as a photocatalyst, even with a small volume of TiO_2_ (15 mm × 15 mm × c.a. 1.5 µm in thickness). The use of deep UV light (λ = 222 nm) as a light source was necessary to enhance photocatalytic activity, due to photo-excitation occurring in both BDD and TiO_2_.

## 1. Introduction

Water shortages are becoming urgent issues, owing to population expansion and rapid economic growth in rising/developing countries. In such countries, the infrastructure for water purification is often inadequate and people may be forced to use polluted water. However, installing a conventional water purification system requires a considerable amount of time and investment, which has prevented uptake. Therefore, there is a need for easily installable, simple, and inexpensive water purification systems.

Photocatalysts are a low cost environmental clean-up technology, which are already widely used in air purification. However, photocatalytic water purification is more difficult (compared to air purification), because of its low efficiency. One reason for this low efficiency is that the light intensity at the photocatalyst surface is weakened by the initial absorption of light by water, lowering its intensity. Another reason is that molecules diffuse slower in water, which decreases the effectiveness of adsorption of pollutants and desorption of decomposed molecules.

To overcome these drawbacks highly efficient photocatalysts are needed, and several approaches to develop such photocatalysts have been tested. One method involves introducing porosity into the photocatalyst [1,2], with the aim of increasing the reactive surface area. In mesoporous Nb_2_O_5_ photocatalysts, crystallites are exposed to mesopores, which promotes access to the active sites. Photocatalytic activity can be enhanced by optimizing the porous structure [1]. Hybridization of nonporous photocatalyst nanoparticles with porous ceramic nanoparticles is another effective way of introducing porosity. Nanocomposites with TiO_2_ nanoparticles and mesoporous SiO_2_ nanoparticles can adsorb dyes more effectively, owing to their large surface areas. These structural features make the initial reaction rates of nanocomposites faster than those of nonporous TiO_2_ [2].

An alternative approach is to form p–n hetero-interfaces. When a p–n heterojunction is created with appropriate energetics, band bending takes place at the p–n heterojunction (to maintain the Fermi level position), and an energy gradient is formed. This gradient promotes photocarrier separation (i.e., suppresses photocarrier recombination), increasing the photocatalytic activity. Notably, hybridization of an n-type metal oxide semiconductor (ZnO [3], TiO_2_ [4,5]) with p-type boron doped diamond (BDD) has produced catalysts with high photocatalytic activity [3,4,5]. Nanostructuring has also been used to increase the reactive surface area and provide an electric path for photogenerated carriers to reach reaction surfaces [3,5].

In this study, we fabricated mesoporous TiO_2_ thin film by a surfactant-assisted sol-gel method, in which we used self-assembly of amphiphilic surfactant micelles as an organic template [6,7] on the BDD layer. The large surface area of the mesopores and promotion of photocarrier separation at p–n heterojunction improved the photocatalytic activity of our TiO_2_ catalysts. Furthermore, we used deep UV light (λ = 222 nm) as a light source. The band gap of BDD is 5.5 eV, and so electron transitions from the valence band (VB) to the conduction band (CB) occur when irradiated by deep UV light with a wavelength shorter than 225 nm. In previous studies, an Hg lamp with a peak excitation at 365 nm was used as a light source [4]. This light source can promote electronic transitions in TiO_2_, which has band gaps of 3.0 and 3.2 eV for rutile and anatase, respectively, although it has less effect on BDD. Therefore, previous studies have not used BDD to its full potential as a photocatalyst. In this study, we aimed to increase the number of photocarriers through photo-excitation of both TiO_2_ and BDD.

## 2. Results and Discussions

**Figure 1:**
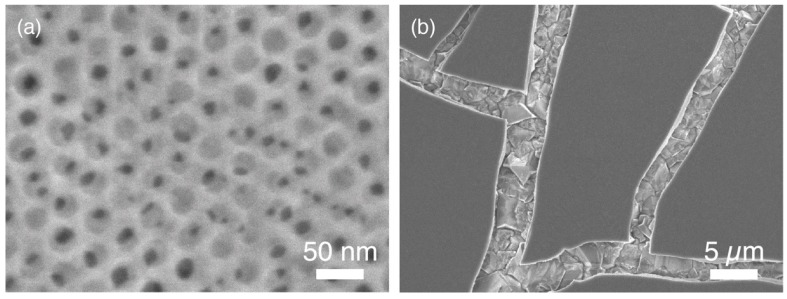
(**a**) High-magnification and (**b**) low-magnification top-view SEM images of the synthesized mesoporous TiO_2_ thin film on the boron doped diamond (BDD) layer.

Figure 1 shows a top-view scanning electron microscope (SEM) image of the synthesized TiO_2_ film. As seen in the previous studies [8,9], hexagonally packed mesopores approximately 20 nm in diameter formed (Figure 1a). However, unlike previous studies, we observed several cracks in the low magnification images (Figure 1b). There are several possible reasons for this cracking. First, the roughness of the underlying BDD layer: As shown in Figure S1, the BDD layer was not smooth. Thus, the precursor solution was unevenly coated onto the BDD layer in the synthesis process. Even if the as-prepared film was coated evenly, the calcination process might also cause breakage of the film. Shrinkage in the vertical direction of films typically occurs during the calcination process [10,11]. Compared with our previous studies in which the film thickness was several hundred nm, the thickness of these as-prepared films was much thicker (i.e., on the order of µm). Therefore, the strain applied to the film during the calcination process was more pronounced. Furthermore, because diamond has a higher thermal conductivity (1000–2200 Wm^−1^K^−1^) and a lower thermal expansion coefficient (1.18 × 10^−6^ K^−1^) than Si (thermal conductivity and thermal expansion coefficient at room temperature of 153 Wm^−1^K^−1^ and 3.8 × 10^−6^ K^−1^, respectively) [12], a large thermal/mechanical stress acts on the TiO_2_/BDD interface during the calcination process. However, exposure of the BDD layer through these cracks is important for photocatalyst applications. Because deep UV light (λ = 222 nm) can reach the BDD surface, photo-excitation in BDD, as well as TiO_2_, becomes possible.

**Figure 2:**
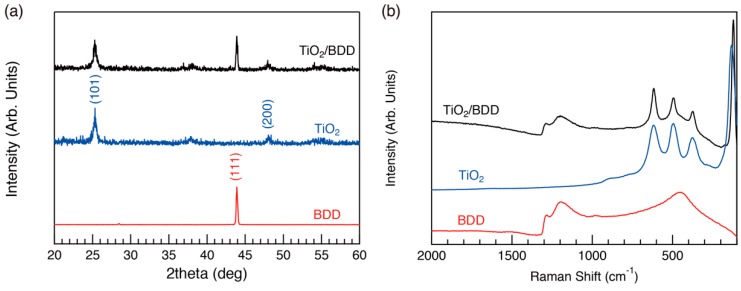
(**a**) Wide-angle X-ray diffraction (WAXD) and (**b**) Raman spectrum of mesoporous TiO_2_/BDD. As a reference, spectra of TiO_2_ (anatase) and BDD are also included.

**Figure 3:**
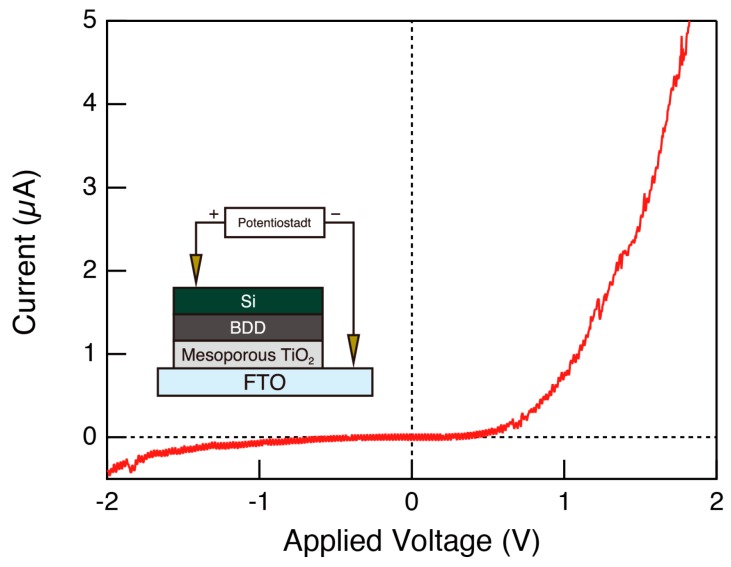
I–V characteristics of mesoporous TiO_2_/BDD heterojunction. Inset shows the schematic illustration of the experimental setup.

We used wide-angle X-ray diffraction (WAXD) to examine the crystallinity of the TiO_2_ framework spectra (Figure 2a) and Raman spectra (Figure 2b). Several diffraction peaks derived from the anatase phase of TiO_2_ and BDD layer appeared in the prepared sample, revealing that the framework of the porous TiO_2_ layer was crystalline. We examined the electrical properties of the mesoporous TiO_2_/BDD, and their I–V curves are shown in Figure 3. Rectifying properties were clearly observed, indicating the formation of a p–n heterojunction.

**Figure 4:**
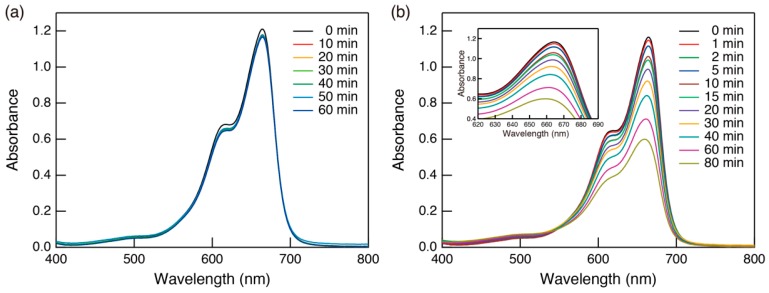
Visible absorption spectra of methylene blue (MB) aqueous solutions sampled from the solution containing mesoporous TiO_2_/BDD at various times (**a**) in the dark and (**b**) during photocatalytic activity test under 222 nm UV light.

**Figure 5:**
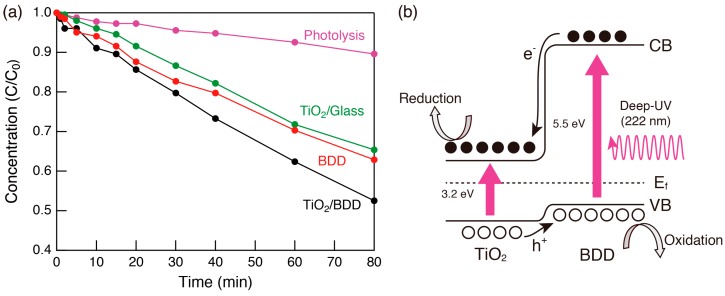
(**a**) Time dependence of the MB concentration, as estimated from the absorption spectra during the photocatalytic activity testing of mesoporous TiO_2_/BDD under 222 nm. For comparison, results of mesoporous TiO_2_/glass, BDD substrate, and photolysis are also included. C and C_0_ are the remaining and initial concentrations of MB, respectively. (**b**) Energy diagram of the mesoporous TiO_2_/BDD and transfer mechanism of photocarriers.

We examined the photocatalytic activity for the decomposition of methylene blue (MB) by evaluating changes of the absorption spectra over time. The time course of the absorption spectra of the MB aqueous solution containing mesoporous TiO_2_/BDD is shown in Figure 4. In the dark (Figure 4a), the peak intensity of the MB absorption spectra decreased slightly at first and then remained constant, indicating that the system had reached adsorption equilibrium. After starting light irradiation (Figure 4b), the absorbance of MB constantly decreased with a blue shift of the adsorption maximum (inset of Figure 4b), owing to photocatalytic decomposition of MB molecules [13]. This clarified that the mesoporous TiO_2_ thin film was photocatalytically active, although the volume of TiO_2_ was small (15 mm × 15 mm × c.a. 1.5 µm in thickness). The decrease of the MB concentration over the time course experiment, as determined from the peak intensity of the absorption spectrum, is shown in Figure 5a. Compared with mesoporous TiO_2_ on a glass substrate, the sample on BDD showed superior photocatalytic activity. Because the film thickness of the mesoporous TiO_2_ was approximately the same regardless of the type of substrate, enhanced photocatalytic activity in the mesoporous TiO_2_/BDD was mainly derived from BDD. The effect can be explained as follows: First, as shown in the energy diagram [Figure 5b], the energy gradient was created at a heterojunction and this gradient promoted carrier separation. Second, the irradiation with deep UV (222 nm) light photo-excited both TiO_2_ and BDD. Thus, electron injection from the conduction band of BDD to TiO_2_ was effective, and more active oxygen species (O_2_^−^) formed by the reduction of dissolved oxygen [5]. In addition, photocatalysis on the BDD surface also occurs. As shown in Figure 5a, BDD operates as a photocatalyst under deep UV (222 nm) light. Because some BDD is exposed by cracks (Figure 1b), MB molecules und UV light can reach the BDD surface. Therefore, two parallel processes (photocatalysis on the TiO_2_ surface, and on the BDD surface) occur simultaneously in mesoporous TiO_2_/BDD.

**Figure 6:**
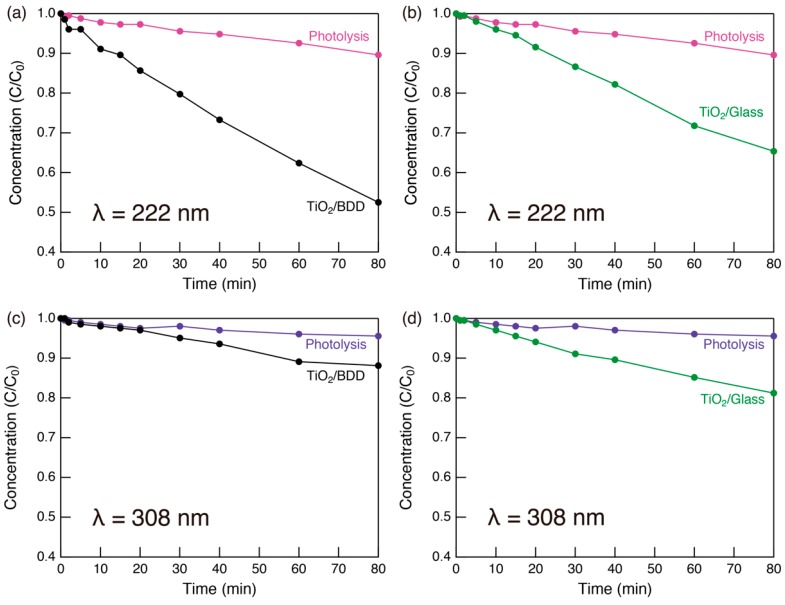
Time dependence of MB concentration, estimated from the visible spectra during the photocatalytic activity testing of (**a**,**c**) mesoporous TiO_2_/BDD and (**b**,**d**) mesoporous TiO_2_/glass under (**a**,**b**) 222 nm and (**c**,**d**) 308 nm UV light.

To clarify the effectiveness of the deep UV light (222 nm) source, we studied the wavelength dependence of photocatalytic activity (Figure 6). When 308 nm UV light was used, no photo-excitation occurred in the BDD as its band gap (ca., 5.5 eV) is much larger than the photon energy (4.0 eV). As shown in Figure 6c, the photocatalytic activity of the mesoporous TiO_2_/BDD decreased, because electrons in the valence band of BDD and electrons in deep traps in the valence band of TiO_2_ cannot be photoexcited (Figure S2). Importantly, there was little difference in the photocatalytic activity of mesoporous TiO_2_/BDD and mesoporous TiO_2_/glass under 308 nm UV irradiation (Figure 6c,d). Thus, the heterojunction only improved effectiveness of the catalyst when photocarriers were excited in BDD. Thus, photo-excitation in BDD and electron injection into TiO_2_ have important influences on the photocatalytic activity.

**Figure 7:**
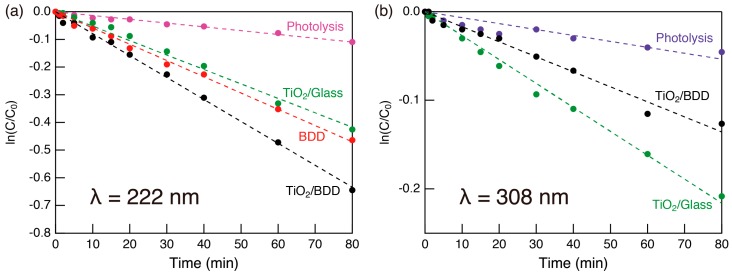
The kinetics of the photocatalytic reaction of MB under (**a**) 222 nm and (**b**) 308 nm UV light. The natural logarithm of C/C_0_ is plotted versus photoirradiation time, where C and C_0_ are the remaining and initial concentrations of MB, respectively. Linear regression lines for calculation of the reaction rate are also included. Note that BDD does not act as a photocatalyst under 308 nm UV light.

The kinetics of the photocatalytic reaction were evaluated from the natural logarithm of the (C/C_0_) as the function of photoirradiation time, assuming that the first-order kinetic expression was applicable (Figure 7). The present system well obeyed the first-order kinetic and, from the slope of the linear regression lines, the reaction rate was calculated and summarized in Table 1. The reaction rate was improved by hybridizing mesoporous TiO_2_ layer and BDD substrate under deep UV (222 nm) light.

## 3. Materials and Methods

### 3.1. Materials

Boron trioxide was purchased from Kanto Chemical Co., Inc. (Tokyo, Japan). The Si(111) wafer was obtained from Matsuzaki Seisakusho Co., Ltd. (Ohda, Shimane, Japan). Diblock copolymer PS(18000)-*b*-PEO(7500) was acquired from Polymer Source Inc. (Dorval, QC, Canada). Titanium chloride, concentrated hydrochloric acid (35–37 wt%), tetrahydrofuran (THF), ethanol, and methylene blue (MB) were purchased from Wako Pure Chemical Industries, Ltd. (Osaka, Japan). These chemicals were used as obtained.

### 3.2. Synthesis of BDD Layer

Boron doped diamond (BDD) thin film (c.a., 7 µm) was synthesized by a chemical vapor deposition (CVD) method, in which boron trioxide was selected as a boron source [14]. A Si (111) wafer was selected as a substrate, and ground with diamond powder for 20 min. The wafer was then cut to a size of 15 mm × 15 mm, and ultrasonically cleaned with acetone, methanol, and distilled water for 10 min each. The concentration of doped boron was set to be 10,000 ppm. The conductivity of the obtained BDD electrode was examined with a resistivity meter Loresta-GX MCP-T700 (Mitsubishi Chemical Analytech (Yamato, Kanagawa, Japan)), and estimated to be approximately 5 × 10^−4^ Ω cm.

### 3.3. Synthesis of Mesoporous TiO_2_ Thin Film on BDD Layer

A mesoporous TiO_2_ thin film on the BDD electrode was prepared according to previous studies [8,9]. The precursor solution was prepared as follows: PS(18000)-*b*-PEO(7500) (50 mg) was dissolved in THF (1.5 mL) at 40 °C. After cooling the solution to room temperature, ethanol (500 µL) was added and the resulting mixture was stirred for 20 min. Separately, titanium chloride (150 µL) was quickly added into concentrated HCl (200 µL). The solution was mixed until the yellow solid intermediate completely dissolved, and then distilled water (300 µL) was added. After stirring for 10 min, this solution was added dropwise to the surfactant solution with stirring for another 30 min. The prepared precursor solution was dropped onto the BDD electrode and spin-coated at 3000 rpm for 30 s. To increase the film thickness, we performed the spin-coating 5 times. Each spin-coated layer was thermally treated at 100 °C for 5 min, before casting the following layer. Finally, the as-prepared film was calcined at 400 °C for 1 h (ramp ratio: 1 °C/min). We confirmed that the calcination process did not affect the conductivity of the BDD electrode in advance. The mesoporous TiO_2_/glass sample was also prepared by the same process, for reference.

### 3.4. Characterization of Mesoporous TiO_2_ Thin Film

We examined morphological features with a scanning electron microscope (SEM) JSM-7600F (JEOL). The crystal structure was examined with an X-ray diffractometer D8 Discover (Bruker) and laser Raman spectrometer NRS-5100 (JASCO). In wide-angle X-ray diffraction (WXRD) measurements, X-rays were irradiated at a very small grazing angle to the synthesized thin film surface, to suppress the background signal from the substrate. Current–voltage (I–V) measurements were performed on a VersaSTAT3 (AMETEK) with manual probes. In this measurement, the mesoporous TiO_2_ layer was placed on transparent conductive fluorine doped tin oxide (FTO) glass substrates (Solaronix (Aubonne, Switzerland)). Manual probes for anode and cathode were placed on the Si (substrate of BDD layer) and FTO, respectively.

### 3.5. Photocatalytic Activity Test

The photocatalytic activity of the obtained thin film was evaluated from decomposition of methylene blue (MB). A 70 mL portion of MB aqueous solution (20 µM) was prepared in a 100 mL glass cup with a Teflon cap. The sample thin film was horizontally placed, in the dark, until adsorption equilibrium was reached. Then, 1.2 mW/cm^2^ of 222 nm UV light from an excimer lamp (ORC Manufacturing Co., Ltd. (Machida, Tokyo, Japan)) was irradiated through the quartz sample tube. The glass cup was capped with a Teflon cap to prevented evaporation during the test, while the MB solution was taken several times by opening the cap. The absorption spectrum of the MB solution was measured with a UV-VIS spectrometer V-670 (JASCO). During the test, the MB solution was constantly stirred. The experimental setup is illustrated in Figure S3. To examine the wavelength dependence, another excimer lamp (ORC Manufacturing Co., Ltd.) emitting 308 nm UV light at 1.2 mW/cm^2^, was also used.

## 4. Conclusions

TiO_2_ thin films with well-ordered mesopores and framework crystallized in the anatase phase were synthesized on a p-type BDD layer. The synthesized mesoporous TiO_2_/BDD had a well-formed p–n heterojunction and operated as an efficient photocatalyst. The use of deep UV light (λ = 222 nm) as a light source increased the photocatalytic efficiency, as photo-excitation in BDD increased the number of photocarriers available to produce active species. As BDD operates as an electrode, we expect that photoelectrochemical water purification may be achieved by application of an electric bias. These studies are now underway, and will be reported in the near future.

## 5. Patents

The contents of this study are the subject of a Japanese Patent application (Application No. 2017-124255).

## Figures and Tables

**Table 1:** Estimated reaction rates (min^−1^) in various conditions.

	222 nm	308 nm
Photolysis	1.37 × 10^−3^	6.71 × 10^−4^
TiO_2_/Glass	5.22 × 10^−3^	2.70 × 10^−3^
TiO_2_/BDD	7.93 × 10^−3^	1.70 × 10^−3^
BDD	5.88 × 10^−3^	Does not act as a photocatalyst

## Supplementary Materials

The following are available online, Figure S1: (**a**) Top- and (**b**) cross-sectional views of the BDD layer, Figure S2: Energy diagram and transfer mechanism of photocarriers in mesoporous TiO_2_/BDD and TiO_2_/glass under 222-nm and 308-nm UV irradiation, Figure S3: Schematic illustration of the experimental setup for photocatalytic activity testing.
