# Supplementary material for: Synthesis of Mesoporous TiO2/Boron-Doped Diamond Photocatalyst and Its Photocatalytic Activity under Deep UV Light (λ = 222 nm) Irradiation
Source: Molecules. 2018 Nov 27;23(12):3095. doi: 10.3390/molecules23123095 (PMC6321498; doi:10.3390/molecules23123095)
Supplement: Supplementary file 1 [file molecules-23-03095-s001.pdf]

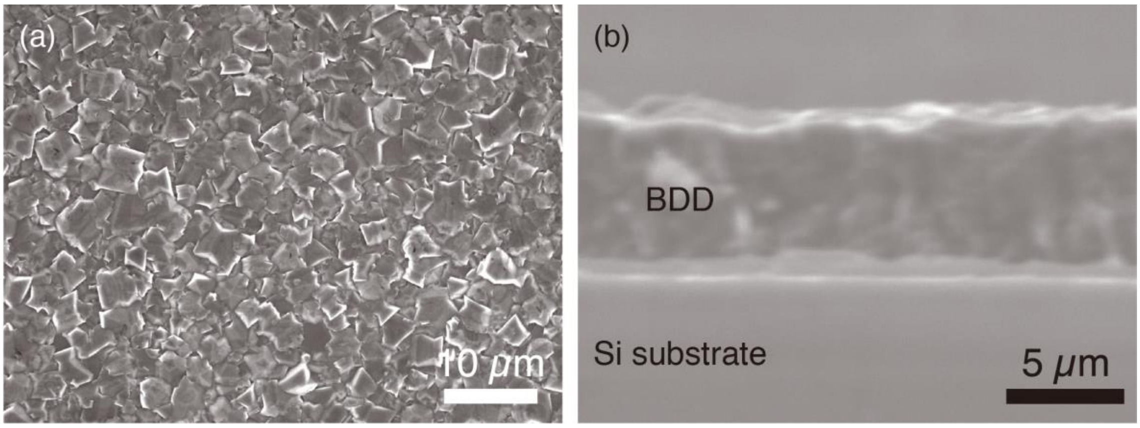

Figure S1. (**a**) Top- and (**b**) cross-sectional views of the BDD layer.

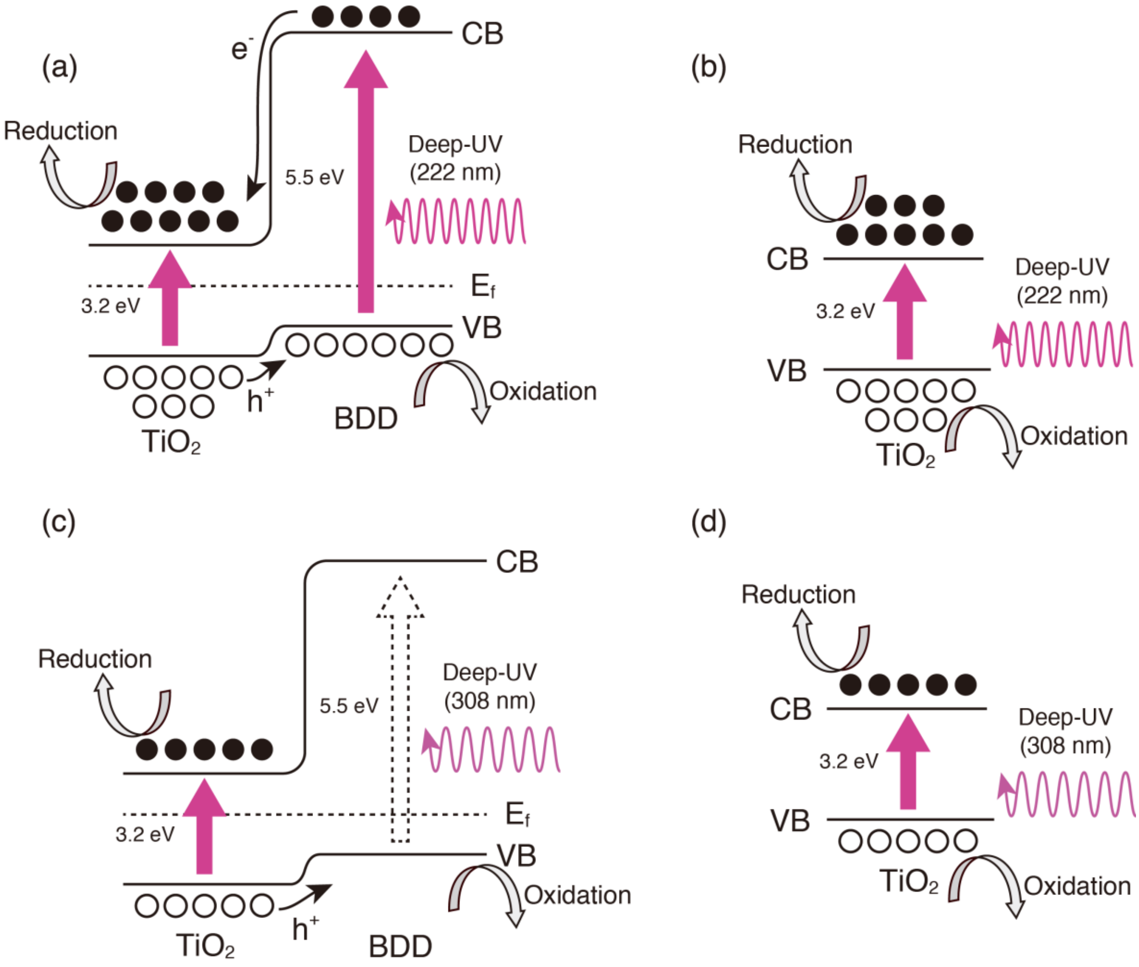

Figure S2. Energy diagram and transfer mechanism of photocarriers in (**a**,**c**) mesoporous TiO_2/BDD and (**b**,**d**) TiO_2/glass under (a,b) 222 nm and (c,d) 308 nm UV irradiation.

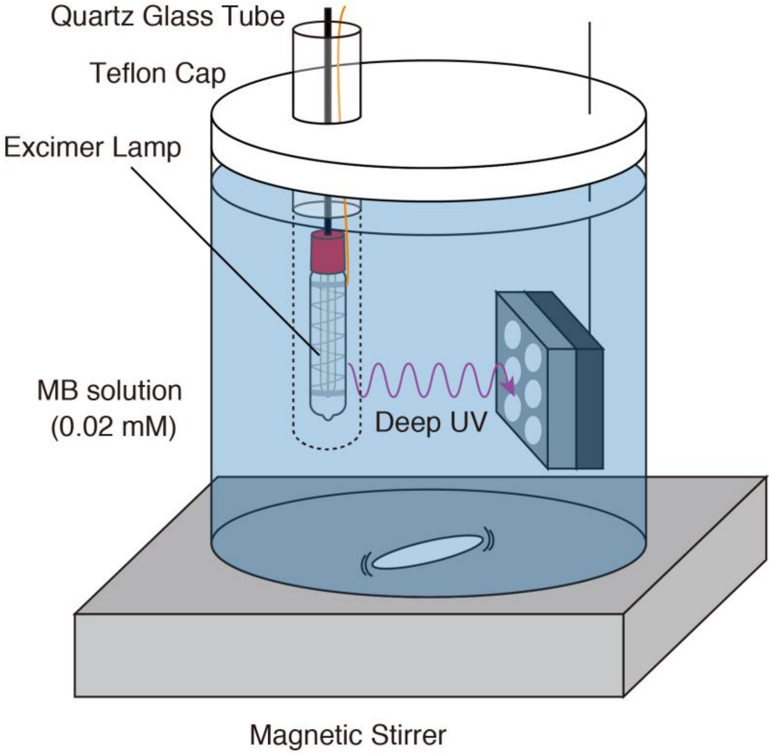

Figure S3. Schematic illustration of the experimental setup used for photocatalytic activity testing.
